# DPD status and fluoropyrimidines-based treatment: high activity matters too

**DOI:** 10.1186/s12885-020-06907-0

**Published:** 2020-05-18

**Authors:** Emmanuel Chamorey, Eric Francois, Marie-Christine Etienne, Jean-Marc Ferrero, Frederic Peyrade, Emmanuel Barranger, Alexandre Bozec, Rémy Largillier, Ophelie Cassuto, Julien Viotti, Renaud Schiappa, Gérard Milano

**Affiliations:** 1 Centre Antoine-Lacassagne, Epidemiology and Biostatistics Unit, Université Côte d’Azur, 33 Avenue de Valombrose, 06189 Nice Cedex 2, Nice, France; 2 Centre Antoine-Lacassagne, Medical Oncology, Université Côte d’Azur, Nice, France; 3 Centre Antoine-Lacassagne, Oncopharmacology Unit, Université Côte d’Azur, Nice, France; 4 Centre Antoine-Lacassagne, Surgical Oncology, Université Côte d’Azur, Nice, France; 5 Centre Antoine Lacassagne, Institut Universitaire de la Face et du Cou, Head and Neck Surgery, Université Côte d’Azur, Nice, France

**Keywords:** Dihydropyrimidine dehydrogenase, Capecitabine, 5-fluorouracil, Digestive neoplasms, Breast neoplasms, Head and neck neoplasms

## Abstract

**Background:**

Dihydropyrimidine dehydrogenase (DPD) status is an indicator of a marked risk for toxicity following fluoropyrimidine (FP)-based chemotherapy. This notion is well-established for low DPD status but little is known about the clinical impact of high DPD activity. This study examined the possible link between high intrinsic lymphocytic DPD activity and overall survival, progression free survival and response to FP-based treatment in patients treated in our institution.

**Methods:**

Lymphocytic DPD activity was assessed in a group of 136 patients receiving FP-based chemotherapy from 2004 to 2016. There were 105 digestive (77.2%), 24 breast (17.6%) and 7 head and neck cancers (5.2%). Cox or logistic regression models were applied with adjustment on all confounding factors that could modify OS, PFS or response. All models were stratified on the three cancer locations. A cut-off for DPD activity was assessed graphically and analytically.

**Results:**

An optimal cut-off for DPD activity at 0.30 nmol/min/mg protein was identified as the best value for discriminating survivals and response. In multivariate analysis, individual lymphocytic DPD activity was significantly related to overall survival (*p* = 0.013; HR: 3.35 CI95%[1.27–8.86]), progression-free survival (*p* < 0.001; HR: 3.15 CI95%[1.75–5.66]) and response rate (*p* = 0.033; HR: 0.33 CI95%[0.12–0.92]) with a marked detrimental effect associated with high DPD activity.

**Conclusions:**

DPD status screening should result in a two-pronged approach with FP dose reduction in case of low intrinsic DPD and, inversely, an increased FP dose for high intrinsic DPD. In a context of personalized FP-based treatment, this innovative strategy needs to be prospectively validated.

## Background

Fluoropyrimidines (FP) are antimetabolite drugs widely used for treatments of many cancers, including digestive, breast, and head and neck locations. FP are delivered as intravenous 5-FU or orally with capecitabine which is a prodrug converted to 5-FU inside target cells. The estimated frequency of significant FP-related toxicity is around 10% including 0.1 to 1.0% of lethal ones [1]. Dihydropyrimidine dehydrogenase (DPD) is the main enzyme responsible for FP catabolism. Population studies [2, 3] on lymphocyte DPD distribution indicate a Gaussian curve of DPD activity distribution including both low and high values. The elucidation of genetic mechanisms underlining DPD variability is increasingly being appreciated [4]. Loss of DPD activity evidenced by phenotypic and/or genotype screening has proven its predictive clinical value in identifying patients at risk for toxicity [1, 3, 5–9]. A recent multicenter prospective study has shown the feasibility of DPYD genotype-guided dose individualization before FP treatment to reduce toxicity [10].

High intrinsic DPD activity results in an accelerated FP degradation [11]. This may translate into a variable marked loss of antitumor efficacy due to the relative lack of FP available for the activation routes at target cell level, and also into a pharmacokinetic effect with a relative decrease in circulating FP [11–14]. The clinical impact of high intrinsic DPD activity on FP-based treatment outcome remained to be investigated. This retrospective monocentric study aimed to fill this gap and examined the link between lymphocytic DPD activity and overall survival, progression-free survival and response rate in a cohort of FP-treated representative patients tested for DPD activity and treated by FP-based chemotherapy in a single institution from 2004 to 2016. The covered cancer locations (digestive tract, breast, head and neck) correspond to the true life of FP-based treatment and were considered in the present study in order to be representative of the routine use of FP in cancer treatment.

## Methods

### Patients

One hundred and thirty-six patients treated by FP-based chemotherapy and assessed for intrinsic DPD activity were included in this retrospective monocentric cohort study covering a twelve-year period from 2004 to 2016. The study comprised of 78 females (57.3%) and 58 males (42.6%); 105 digestive cancers (77.2%), 24 breast cancers (17.6%) and 7 head and neck cancers (5.2%). All patients were consecutively treated at the Centre Antoine Lacassagne (Comprehensive Cancer Center, Nice, France). Intrinsic DPD activity (nmol/min/mg protein) in peripheral blood mononuclear cells was assessed as previously described by Harris and coll [15, 16].. In brief, DPD activity (blood sample taken between 9:00 and 11:00 am) was measured in mononuclear cells using a radioenzymatic assay (2.5 mM MgCl2, 250 μM NADPH, 20 μM 14C5-FU) with separation of 14C5-FU from 14C5-FUH2 by High-Performance Liquid Chromatography (HPLC) coupled with a radiodetector. DPD testing was performed before start of FP-treatment or after initiation of FP-treatment. This study was declared to the Commission Nationale Informatique et Liberté (CNIL N°17,002) and all patients agreed that their data could be used for retrospective biomedical study.

### Statistics

OS was defined as the time from FP initiation to death. PFS was defined as the time from FP initiation to death or either distant, local or metastatic progression as defined by the RECIST version 1.1 criteria. Patients showing no event (death or progression) or lost to follow-up were censored at the date of their last contact. In our study, response to therapy was evaluated prospectively using RECIST version 1.1 criteria’s when possible. Otherwise, observed complete response during FP treatment (CR) was defined as disappearance of all target lesions after chemotherapy alone or multimodal treatment including chemotherapy, radiotherapy and/or local ablative therapy. Statistical comparisons were performed using Cox regression model for survival data or logistic regression model for response analysis. Others quantitative data were compared by using Student T test or Wilcoxon test when appropriated and qualitative data were analyzed by using Fisher exact test. In order to avoid all confounding factors that could modify OS, PFS or Response, all multivariate models were stratified for cancer locations (digestive, breast and head and neck) and adjusted for all variables associated with *p* < 0.10 on univariate analysis. Variables analyzed were defined as follow: DPD activity (nmol/min/mg protein), sex (male/female), disease severity (local/advanced), DPD prescription before FP start or during FP treatment, other chemotherapy treatment before FP (no/yes), type of FP-based treatment (Capecitabine, 5-FU, Both alternatively), observed FP-based toxicity > grade 3 or 4 by using CTCAE V4.0 or toxicity inducing FP-based treatment stop (no/yes), any surgery associated to FP (no/yes), any radiotherapy associated to FP (no/yes), observed complete response during FP treatment (no/yes), any local, loco regional or metastatic recurrence observed (no/yes), age at FP-based treatment (years), number of chemotherapy lines including FP treatment (N), number of FP cycles (N). Proportional hazards were tested for all variables entered in Cox models using graphical Schoenfeld residuals and statistical test. Sensitivity analyses for all tested models were performed firstly by removing outliers when indicated and secondly by testing the same multivariate models only for the 105 digestive cancer patients. Statistical analyses were two-sided and were performed using R-3.2.3 for Windows.

### DPD cut-off assessment

The cut-off for DPD activity predicting OS, PFS and response was evaluated graphically by using inflection points of the smoothing spline curve fit to DPD activity (see Figs. S1, S2 and S3 in supplementary data showing smoothing spline fit to DPD activity versus OS, PFS and Response) and confirmed by using statistical rules assessed using R function “bestcut2” for survival data model as well as the R function “optimal.cutpoints” which established the optimal cut-point for a logistic regression model.

## Results

DPD activity followed a Gaussian distribution with mean (+/−SD) at 0.21 nmol/min/mg (+/− 0.10) and quartiles [0.002–0.15-0.20-0.28-0.48]. The best identified cut-off for DPD activity predicting OS, PFS and response was 0.30 nmol/min/mg protein; this DPD value was included in all univariate and multivariate analysis. This cut-off is the most relevant point according to graphical and analytical assessment and is corresponding to almost the third quartile of the distribution of DPD activity (i.e.: 0.28 nmol/min/mg protein). Twenty-five patients (18.4%) presented DPD activity ≥0.30 and 111 (81.6%) presented DPD activity < 0.30 nmol/min/mg protein. Most of the DPD activity assessments were performed before the beginning of FP-based treatment (108; 79.4%). No difference was observed in DPD activity when measurement was performed before or during FP-based treatment (*p* = 0.59, Table 1). Ninety six patients (70.5%) had advanced disease, including 66 with loco-regional and 30 with metastatic disease. Mean age was 64.2 years (+/− 12.4). Thirty-eight patients (27.9%) received capecitabine based treatment, 70 (51.5%) received 5-Fluorouracil based treatment (5-FU) associated with folinic acid and 28 (20.6%) received both treatments alternatively. Median number of total FP cycles was 8 (range: 1–48); 33 patients (24.3%) received other chemotherapy prior to FP-based treatment. Median number of chemotherapy lines (FP or not FP) was 1 (range 1–12). One hundred patients (73.5%) had surgery associated with FP-based treatment and 77 (56.6%) received radiotherapy. Seventy-six patients (55.9%) showed complete response during FP-based treatment. Recurrence was observed in 77 (56.6%) patients and 58 patients (42.6%) presented FP-related toxicity. Table 1 brings more detailed information regarding patient and treatment characteristics versus DPD activity levels (low versus high, ie: < 0.30 versus ≥0.30) it shows that observed complete response during FP treatment (*p* = 0.032, Fisher exact test), was significantly linked to DPD activity.

**Table 1:** Patient and treatment characteristics versus DPD activity levels

Variable	Modality	Low DPD activity (< 0.30 nmol/min/mg prot)	High DPD activity (≥ 0.30 nmol/min/mg prot)	Fisher-test
Age at FP-based treatment (NA = 0)	Mean [SD]	64.22 [12.11]	64.38 [13.67]	0.95*
Sex (NA = 0)				0.17
	Female	67 (85.9%)	11 (14.1%)	–
	Male	44 (75.86%)	14 (24.14%)	–
Cancer location (NA = 0)				0.76
	Digestive	86 (81.9%)	19 (18.1%)	–
	Head and Neck	5 (71.43%)	2 (28.57%)	–
	Breast	20 (83.33%)	4 (16.67%)	–
Disease severity (NA = 0)				0.33
	Local disease	35 (87.5%)	5 (12.5%)	–
	Advanced disease	76 (79.17%)	20 (20.83%)	–
DPD prescription (NA = 0)				0.59
	Before FP start	89 (82.41%)	19 (17.59%)	–
	During FP Treatment	22 (78.57%)	6 (21.43%)	–
Other chemotherapy before FP (NA = 0)				0.61
	No	85 (82.52%)	18 (17.48%)	–
	Yes	26 (78.79%)	7 (21.21%)	–
FP-based treatment (NA = 0)				0.61
	Capecitabine	29 (76.32%)	9 (23.68%)	–
	5-FU	58 (82.86%)	12 (17.14%)	–
	Both alternatively	24 (85.71%)	4 (14.29%)	–
FP-based toxicity (NA = 4)				0.17
	No	57 (52.78%)	16 (69.57%)	–
	Yes	51 (47.22%)	7 (30.43%)	–
Surgery associated to FP (NA = 1)				0.31
	No	31 (88.57%)	4 (11.43%)	–
	Yes	79 (79%)	21 (21%)	–
Radiotherapy associated to FP (NA = 0)				0.65
	No	47 (79.66%)	12 (20.34%)	–
	Yes	64 (83.12%)	13 (16.88%)	–
Nb. of chemotherapy lines (NA = 1)	Median [Min-Max]	1 [1–12]	2 [1–5]	0.26**
Nb. of FP cycles (NA = 0)	Median [Min-Max]	7 [1–48]	9 [1–18]	0.39**
Observed complete response during FP treatment (NA = 6)				0.032
	No	35 (34.31%)	14 (60.87%)	–
	Yes	67 (65.69%)	9 (39.13%)	–
Any recurrence (NA = 0)				0.12
	No	52 (46.85%)	7 (28.0%)	–
	Yes	59 (53.15%)	18 (72.0%)	–

*NA* not available data, *FP* Fluoropyrimidine, *Fisher-test p*-value Fisher exact test, * *p*-value Student-T test, ** *p-*value Wilcoxon test, *SD* Standard Deviation

**Fig. 1:**
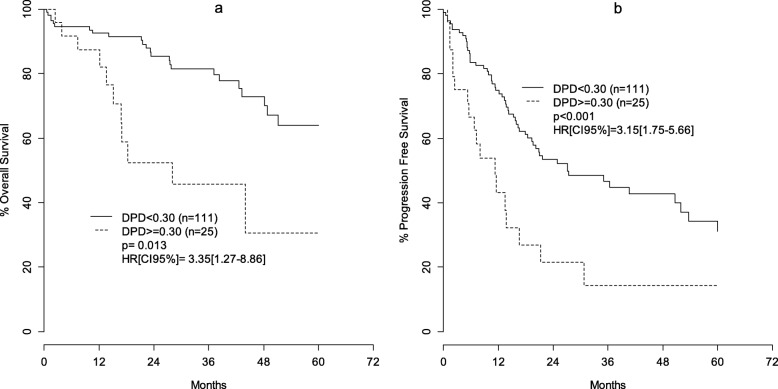
**a** anb **b** Kaplan Meier survival curves for Overall and Progression Free Survival according to low (< 30) and high (≥30) DPD activity nmol/min/mg protein; p: adjusted *p*-value cox model; HR [CI95%]: adjusted hazard ratio [confidence interval 95%]

Univariate analyses for overall survival, progression-free survival and response rate are summarized in Table 2. OS, PFS and response rate were significantly related to DPD activity (*p* = 0.0018, 0.0016 and 0.022, respectively) with, a marked detrimental effect linked to high DPD activity. Survival curves for OS and PFS are depicted in Fig. 1 a and b. Table 2 shows that disease severity (*p* = 0.046), surgery associated to FP (*p* = 0.030), observed complete response during FP treatment (*p* < 0.0001), any recurrence (*p* = 0.0026) and age (*p* = 0.0062) were significantly associated with OS. Disease severity at initial diagnostic (*p* = 0.0079), other chemotherapy before FP (*p* = 0.0022), age (*p* = 0.0069) and number of FP cycle (*p* < 0.001) were significantly related to PFS. Also illustrated from Table 2, disease severity at initial diagnostic (*p* = 0.008) and other chemotherapy before FP (*p* = 0.00069) were found significantly related to response.

**Table 2:** Overall survival, progression free survival and observed complete response univariate analysis

		Overall Survival			Progression free survival			Observed complete response		
**Variable**	**Modality**	**HR**	**CI95%**	**p-Cox**	**HR**	**CI95%**	**p-Cox**	**OR**	**CI95%**	**p-RegLog**
DPD activity	Low < 0.30	1	REF.	–	1	REF.	–	1	–	–
	High ≥0.30	3.1	[1.50–6.50]	0.00188	2.40	[1.40–4.0]	0.00158	0.34	[0.13–0.87]	0.022
Sex	Female	1	REF.	–	1	REF.	–	1	–	–
	Male	1.8	[0.92–3.50]	0.0888	1.4	[0.92–2.3]	0.115	0.87	[0.42–1.80]	0.7
Cancer location	Digestive	1	REF.	–	1	REF.	–	1	–	–
	Head and Neck	1.2	[0.42–3.60]	0.701	0.78	[0.31–2.0]	0.593	0.44	[0.089–2.1]	0.29
	Breast	0.38	[0.14–1.00]	0.0613	0.68	[0.37–1.3]	0.219	0.71	[0.26–1.90]	0.49
Disease severity	Local disease	1	REF.	–	1	REF.	–	1	–	–
	Advanced disease	2.60	[1.00–6.80]	0.0462	2.2	[1.2–3.90]	0.00797	0.29	[0.11–0.74]	0.008
Other chemotherapy before FP	No	1	REF.	–	1	REF.	–	1	–	–
	Yes	1.2	[0.59–2.50]	0.606	2.1	[1.3–3.30]	0.00227	0.22	[0.09–0.54]	0.00069
FP-based treatment	CAP	1	REF.	–	1	REF.	–	1	–	–
	FU	0.90	[0.40–2.00]	0.798	1.5	[0.8–2.60]	0.217	0.83	[0.34–2.00]	0.68
	FU_CAP	0.74	[0.28–1.90]	0.54	1.7	[0.87–3.3]	0.124	0.41	[0.14–1.20]	0.095
FP-based toxicity	No	1	REF.	–	1	REF.	–	1	–	–
	Yes	0.83	[0.41–1.70]	0.597	0.93	[0.58–1.5]	0.759	0.64	[0.31–1.30]	0.23
Surgery associated to FP	No	1	REF.	–	1	REF.	–	1	–	–
	Yes	0.46	[0.23–0.93]	0.0309	0.92	[0.55–1.6]	0.756	1.7	[0.74–3.80]	0.2
Radiotherapy associated to FP	No	1	REF.	–	1	REF.	–	1	–	–
	Yes	0.58	[0.29–1.20]	0.127	0.66	[0.41–1.1]	0.0797	1.2	[0.55–2.40]	0.7
Observed complete response during FP treatment	No	1	REF.	–	–	–	–	–	–	–
	Yes	0.098	[0.037–0.25]	< 0.0001	–	–	–	–	–	–
Any recurrence	No	1	REF.	–	–	–	–	–	–	–
	Yes	5.00	[1.70–14.0]	0.00265	–	–	–	–	–	–
Age at FP-based treatment		1.04	[1.01–1.07]	0.0062	1.03	[1.01–1.05]	0.0069	0.98	[0.95–1.01]	0.21
Number of chemotherapy lines		0.93	[0.80–1.07]	0.31	1.08	[0.99–1.16]	0.065	0.86	[0.74–1.01]	0.06
Number of FP cycles		0.99	[0.96–1.02]	0.56	1.03	[1.02–1.05]	< 0.001	0.98	[0.95–1.01]	0.30

*p-Cox p*-value of Cox regression model, *p-RegLog p*-value regression logistique, *HR* Hazard ratio, *CI95%* confidence interval 95%, *FP* Fluoropyrimidine, *REF* reference value

In multivariate analysis (Table 3) including all variables with *p* < 0.10 in univariate analysis and a stratification for cancer locations (digestive, breast and head and neck), DPD remained a significant strong and independent prognostic factor for OS, PFS and response (*p* = 0.013, HR: 3.35 CI95% [1.27–8.86]; *p* < 0.001, HR: 3.15 CI95% [1.75–5.66] and *p* = 0.033, HR: 0.33 CI95% [0.12–0.92] respectively). Supplementary data (Tables S1 to S3) provide detailed statistical results of multivariate regression analyses for OS, PFS and response and it shows that surgery associated to FP (0.0017) and observed complete response during FP treatment (< 0.001) remained significantly related to OS. It was also put into evidence (Table S2) that other chemotherapy before FP (< 0.001), age at FP-based treatment (0.001) and number of FP cycles (0.006) were significantly associated to PFS. Disease severity (Table S3) at initial diagnostic (0.014) and other chemotherapy before FP (0.0049) were significantly linked to response.

**Table 3:** Univariate and multivariate analysis for overall survival, progression free survival and response according to DPD

Variable	Univariate analysis		Multivariate analysis	
	***p***-value	HR [CI95]	***p***-value	HR [CI95]
**DPD activity versus overall survival**^a^				
< 0.30	–	–	–	–
≥ 0.30	0.0018	3.1 [1.50–6.50]	0.013	3.35 [1.27–8.86]
**DPD activity versus progression free survival**^a^				
< 0.30	–	–	–	–
≥ 0.30	0.0016	2.40 [1.40–4.00]	< 0.001	3.15 [1.75–5.66]
**DPD activity versus response to fluoropyrimidine**^b^				
< 0.30	–			
≥ 0.30	0.022	0.34 [0.13–0.87]	0.033	0.33 [0.12–0.92]

*HR [CI95]* Hazard ratio and confidence interval 95%

^a^ Cox proportional hazards model

^b^ Logistic regression model

All variables associated with *p* < 0.10 in univariate analysis were included in a Cox regression or Logistic regression model. All models were stratified on cancer locations (digestive, breast, head and neck)

Sensitivity analyses to evaluate the stability of multivariate models were tested firstly by removing 6 patient outliers from OS and PFS models and 5 patient outliers from response model. DPD activity remained significant with adjusted *p*-value at 0.001, < 0.0001 and 0.0064 for OS, PFS and response in multivariate models respectively. And in addition, by testing the same multivariate models only for patients with digestive cancer (*n* = 105), DPD activity was still significantly related to OS and PFS (adjusted p-value at 0.019 and 0.0007 respectively) and there was a strong tendency for a link with response (*p* = 0.075).

## Discussion

From the present study it appears that high intrinsic DPD activity (≥0.30 nmol/min/mg protein) is linked to OS, PFS and response in FP-treated patients. This is a well-established point that low DPD activity is a predictive risk factor for FP-associated toxicity [1, 3, 5–9]. A potential benefit of DPYD genotype-guided FP-dose individualization has been recently reported [10]. French Health Autoritaries (HAS) have provided strong recommendations to perform DPD deficiency screening on the basis of uracilemia determination before any treatment by FP with a guidance for dose reductions [17]. European authorities now consider that it is necessary to perform an Eudra Vigilance analysis for reports of DPD-FP-related toxicity. From our results, a new and complementary FP dosing strategy is pointing out. The fact that high individual DPD activity confers a particularly poor survival is new, as presently established in this retrospective series of 136 cancer patients.

Although different treatments regimens used in this study (capecitabine, 5-FU, both) could potentially mitigate the conclusions, all patients received a FP-based therapy. The distribution of DPD activity in the study population followed a Gaussian distribution comparable to those previously published and is thus representative of DPD activity profile in treated patients [2, 3]. Behind the Gaussian shape of DPD activity lies in fact a myriad of impacting mutations [6, 18]. In addition, there is the existence of a regulation of DPD gene expression at a transcriptional level [19, 20]. Of note, as recently been reported, there is the presence of rare mutations which confer high intrinsic DPD activity [1]. Theoretically, germinal DPD status can impact DPD expression at tumoral level. However, the fact that Tp53 has recently been shown to play an important role in controlling pyrimidine catabolism through repression of DPD expression at tumoral level may moderate this view [21]. A marked variability in intra-tumoral DPD expression was previously reported to be linked to the occurrence of resistance to FP therapy, at both experimental [11] and clinical levels [14]. Our previous study [22] indicated that both thymidylate synthase and DPD are overexpressed in deficient mismatch repair dMMR tumours, thus providing possible explanation for a relative resistance of MMR tumours to FP-based therapy [23]. More recently, in a trial randomized to either gemcitabine or 5-FU plus folinic acid in pancreatic carcinoma, Elander et al. [24] showed that high intra-tumoral DPD expression was a negative prognostic biomarker and that low DPD tumor expression indicated better prognosis, at least for patients treated with 5-FU.

Therefore, at least in part, high intrinsic DPD activity (as here measured in PBMC) may reflect DPD activity in tumors and influence disease outcome following FP therapy. It is also clear that the impact of high intrinsic DPD activity is pharmacokinetic by nature with, consequently, relatively low levels of circulating FP resulting from high global DPD activity, a phenomenon occurring in the liver. In line with this view is the previously reported link between patient survival and 5-FU circulating exposure [25, 26]. The existence of a significant correlation between DPD activity in normal liver tissue and lymphocytes [27] supports the extrapolation between lymphocyte DPD activity and FP pharmacokinetic behavior with clinical consequences. Thus, although the present study did not incorporate FU pharmacokinetic analyses, one can estimate that abnormally high DPD activity may be translated into low FP circulating levels, leading to a relative loss of treatment efficacy, at least for iv-FP keeping in mind that capecitabine delivers FU at the target cellular level itself.

Ideally, an intrinsic prognostic role for DPD activity should be tested in patients not receiving FP. This appears justified because it has recently been shown that a nucleotide imbalance exists across different cancer types. This imbalance can potentially be introduced by abnormal DPD activity and a pyrimidine/purine disequilibrium may increase mutagenesis [28, 29] and consequently may influence disease outcome [29].

The present exploratory study has several limitations. This is a retrospective and heterogeneous cohort study including digestive, breast and head & neck FP-treated cancer patients receiving multifactorial treatment like intravenous 5-FU, oral capecitabine or both. To avoid most of the biases in the analyses, all multivariate analysis have been stratified on cancer location and these analyses were also adjusted on all variables that could be confounding factors. Moreover, the same statistically significant results were found when analyses were made on digestive cancer patients only.

## Conclusions

**Fig. 2:**
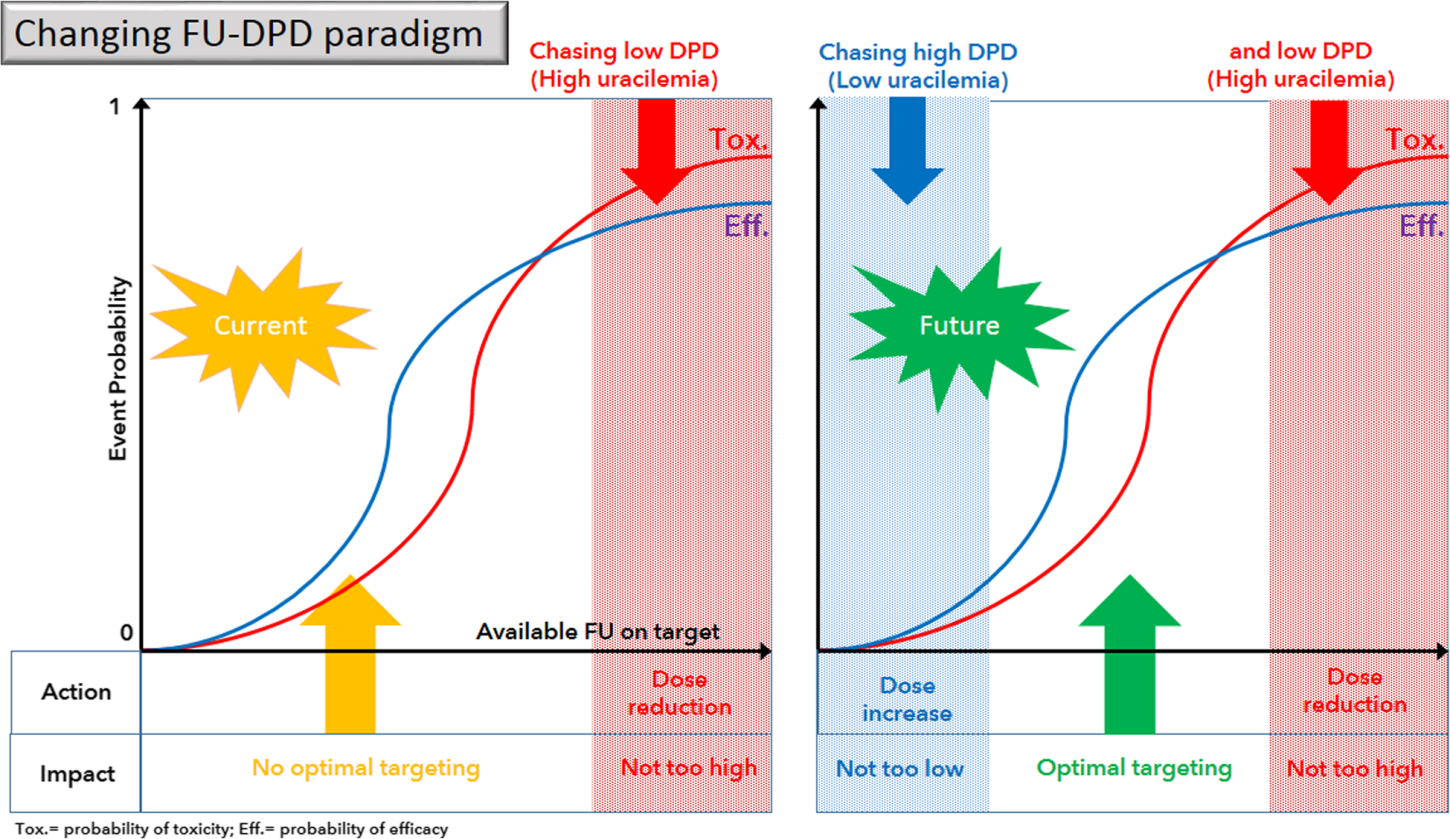
Changing FU-DPD paradigm to optimizing FU-based treatment; abscissa = available FU on target; ordered = probability of event; Tox. = probability of efficacy; Eff. = probability of toxicity; left side = current paradigm, right side = future paradigm taking into account our results

Above all, the present report may confer practical implications in the current era of precision medicine in cancer. Hence, at the light of the present data, DPD activity screening could result in a two-pronged approach: FP dose reduction in case of low intrinsic DPD activity and, inversely, an increased FP dose for high intrinsic DPD activity (Fig. 2). This view contrasts with the caricatured current strategy consisting in reducing FP dosage in cases of DPD abnormality (on a phenotypic and/or a genotypic basis) potentially diminishing the ability to clear 5-FU from the body [18]. To ignore cases with relatively high intrinsic DPD activity could confer a risk for a loss of treatment success. In final, the present study contributes to broaden the strategy regarding DPD screening. Such a new strategy needs to be prospectively validated for setting a true personalized DPD-based treatment including pharmacokinetics analyses. In line with this view, our group is currently conducting a multicenter clinical study (DPD-MAX trial N° Eudract 2017–002037-31) aiming at establishing a more reliable prospective validation of the link between high intrinsic DPD activity and loss of FP treatment efficacy.

## Supplementary information

**Additional file 1: Table S1.** Overall survival multivariate analysis. **Table S2.** Progression free survival multivariate analysis. **Table S3.** Observed complete response during FP treatment multivariate analysis.

**Additional file 2: Fig. S1.** Smoothing spline and CI95% fit to DPD activity nmol/min/mg protein versus overall survival and 95% confident interval. **Fig. S2.** Smoothing spline fit and CI95% to DPD activity nmol/min/mg protein versus progression free survival and 95% confident interval. **Fig. S3.** Smoothing spline and IC95% fit to DPD activity nmol/min/mg protein versus observed complete response during FP treatment.

## Data Availability

The datasets during and/or analysed during the current study available from the corresponding author on reasonable request.

## Abbreviations

DPD: DihydroPyrimidine Dehydrogenase
FP: FluoroPyrimidine
5-FU: 5 Fluorouracyl
CI95%: 95% Confident Interval
HPLC: High-Performance Liquid Chromatography
CR: Complete Response
HAS: French Health Autoritaries
OS: Overall Survival
PFS: Progression Free Survival
PBMC: Peripheral Blood Mononuclear Cell
HR: Hazard Ratio
