# Supplementary material for: DPD status and fluoropyrimidines-based treatment: high activity matters too
Source: BMC Cancer. 2020 May 18;20:436. doi: 10.1186/s12885-020-06907-0 (PMC7236295; doi:10.1186/s12885-020-06907-0)
Supplement: Supplementary file 1 — Additional file 1: Table S1. Overall survival multivariate analysis. Table S2. Progression free survival multivariate analysis. Table S3. Observed complete response during FP treatment multivariate analysis. [file 12885_2020_6907_MOESM1_ESM.docx]

**SUPPLEMENTARY DATA (TABLES)**

Table S1: Overall survival multivariate analysis

|  | **HR** | **[CI95%]** | **p-Cox** |
| --- | --- | --- | --- |
| DPD activity | 3.35 | [1.27-8.86] | 0.013 |
| Sex | 1.37 | [0.53-3.55] | 0.517 |
| Disease severity at initial diagnostic | 2.42 | [0.68-8.54] | 0.171 |
| Surgery associated to FP | 0.19 | [0.07-0.54] | 0.0017 |
| Observed complete response during FP treatment | 0.10 | [0.03-0.33] | <0.001 |
| Any recurrence | 3.07 | [0.65-14.5] | 0.157 |
| Age at FP-based treatment | 1.03 | [1.00-1.07] | 0.064 |

p-Cox : p-value of Cox regression model; HR: adjusted Hazard ratio; [CI95%] : confidence interval 95%; FP: Fluoropyrimidine

All multivariate analysis were stratified on cancer location (Digestive, Head and Neck, Breast)

Table S2: Progression free survival multivariate analysis

|  | **HR** | **[CI95%]** | **p-Cox** |
| --- | --- | --- | --- |
| DPD activity | 3.15 | [1.75-5.66] | <0.001 |
| Disease severity at initial diagnostic | 1.81 | [0.97-3.39] | 0.064 |
| Other chemotherapy before FP | 4.71 | [2.2-10.08] | <0.001 |
| Radiotherapy associated to FP | 0.93 | [0.51-1.69] | 0.817 |
| Age at FP-based treatment | 1.05 | [1.02-1.07] | 0.001 |
| Number of chemotherapy lines | 1.03 | [0.84-1.26] | 0.79 |
| Number of FP cycles | 1.04 | [1.01-1.07] | 0.006 |

p-Cox : p-value of Cox regression model; HR: adjusted Hazard ratio; [CI95%] : confidence interval 95%; FP: Fluoropyrimidine

All multivariate analysis were stratified on cancer location (Digestive, Head and Neck, Breast)

Table S3: Observed complete response during FP treatment multivariate analysis

| **Variables** | **OR** | **[CI95%]** | **p-RegLog** |
| --- | --- | --- | --- |
| DPD activity | 0.33 | [0.12-0.92] | 0.033 |
| Disease severity at initial diagnostic | 0.3 | [0.11-0.83] | 0.014 |
| Other chemotherapy before FP | 0.16 | [0.04-0.62] | 0.0049 |
| Number of chemotherapy lines | 0.9 | [0.68-1.19] | 0.466 |

p-RegLog: p-value logistic regression model; OR: Adjusted Odds ratio; [CI95%] : confidence interval 95%; FP: Fluoropyrimidine

All multivariate analysis were stratified on cancer location (Digestive, Head and Neck, Breast)
