# Supplementary material for: DPD status and fluoropyrimidines-based treatment: high activity matters too
Source: BMC Cancer. 2020 May 18;20:436. doi: 10.1186/s12885-020-06907-0 (PMC7236295; doi:10.1186/s12885-020-06907-0)
Supplement: Supplementary file 2 — Additional file 2: Fig. S1. Smoothing spline and CI95% fit to DPD activity nmol/min/mg protein versus overall survival and 95% confident interval. Fig. S2. Smoothing spline fit and CI95% to DPD activity nmol/min/mg protein versus progression free survival and 95% confident interval. Fig. S3. Smoothing spline and IC95% fit to DPD activity nmol/min/mg protein versus observed complete response during FP treatment. [file 12885_2020_6907_MOESM2_ESM.docx]

**SUPPLEMENTARY DATA (FIGURES)**

Figure S1:

Smoothing spline and CI95% fit to DPD activity nmol/min/mg protein versus overall survival and 95% confident interval


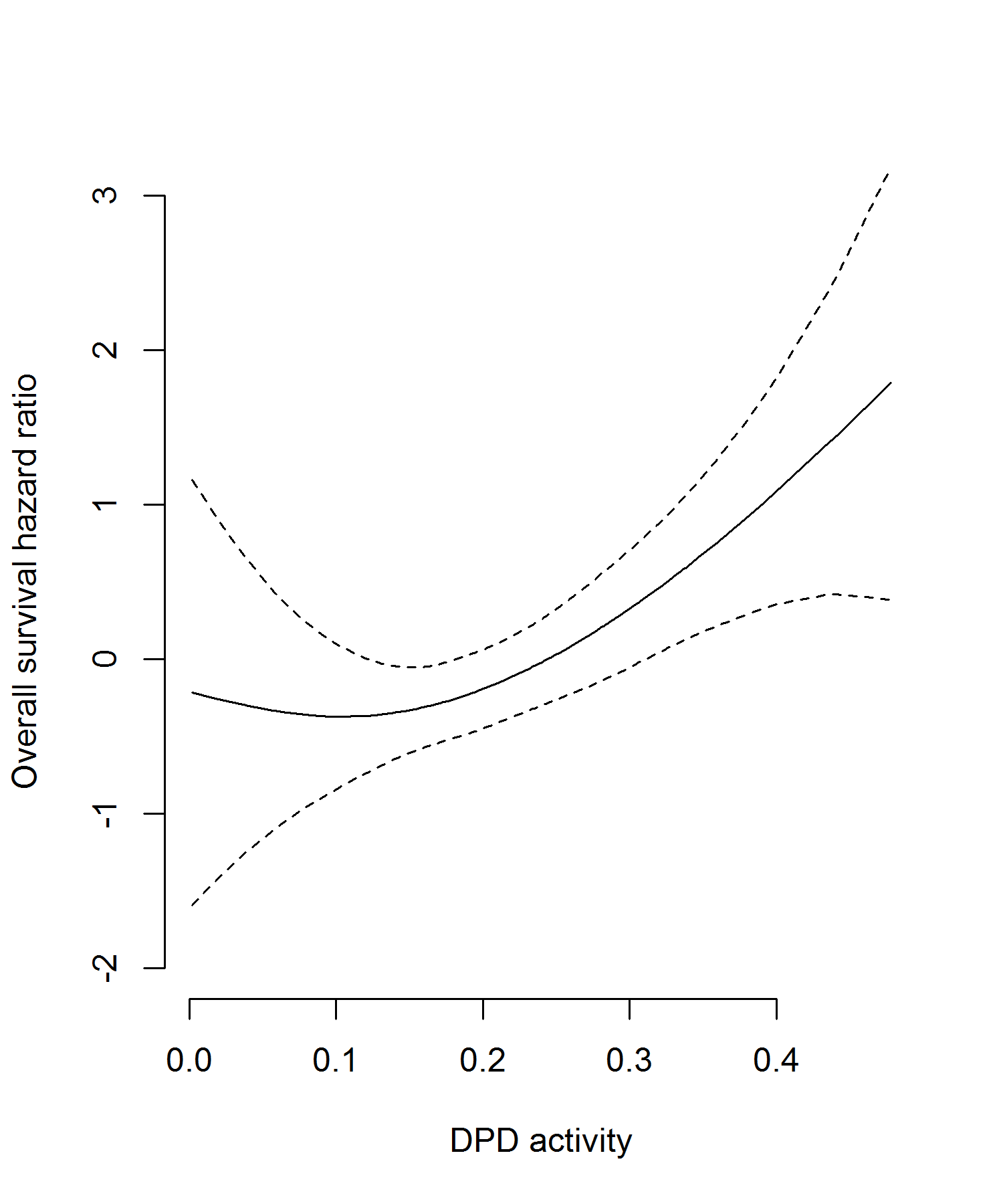


Figure S2:

Smoothing spline fit and CI95% to DPD activity nmol/min/mg protein versus progression free survival and 95% confident interval


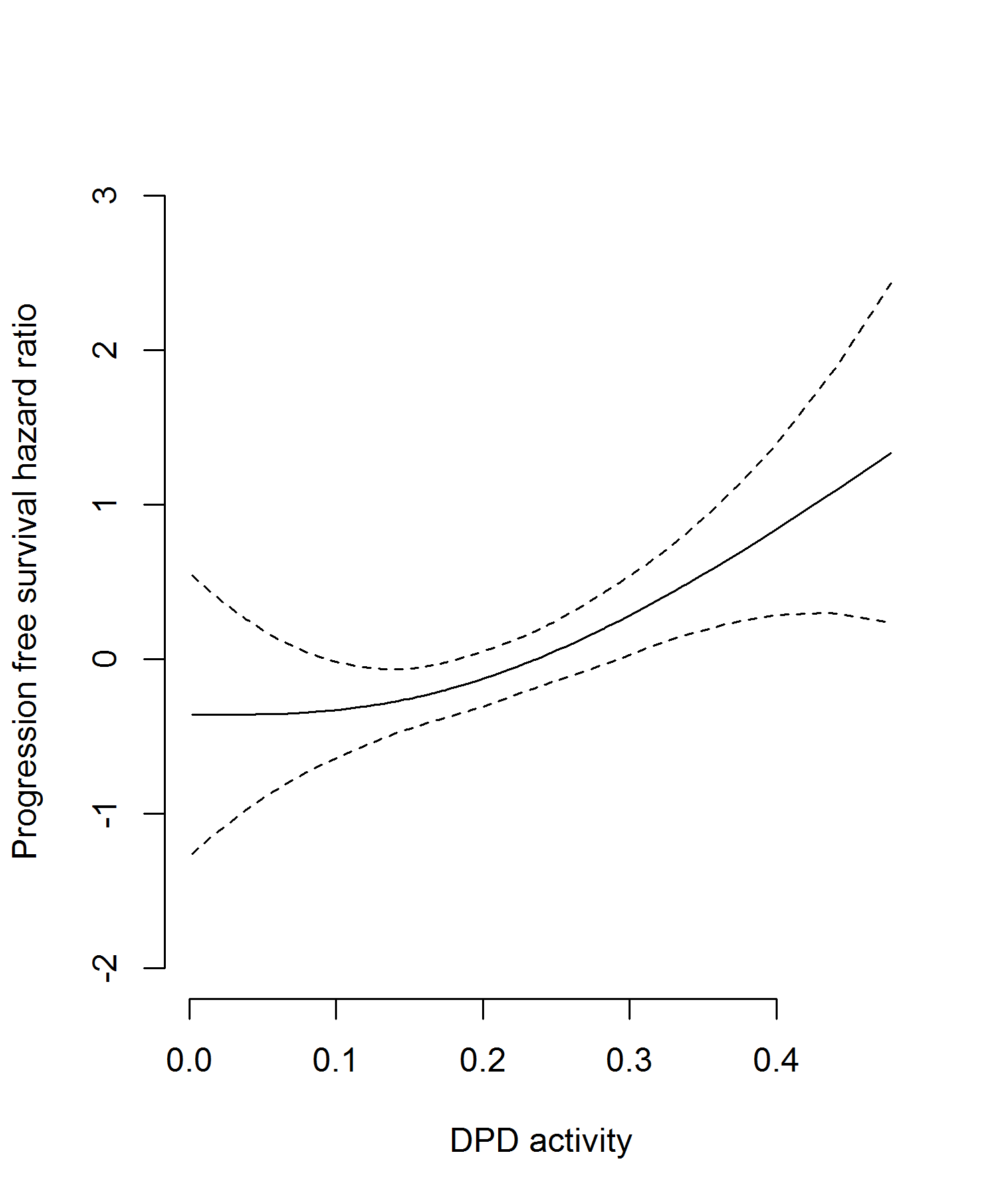


Figure S3:

Smoothing spline and IC95% fit to DPD activity nmol/min/mg protein versus observed complete response during FP treatment
